# Differential innate immune response of endometrial cells to porcine reproductive and respiratory syndrome virus type 1 versus type 2

**DOI:** 10.1371/journal.pone.0284658

**Published:** 2023-04-26

**Authors:** Muttarin Lothong, Dran Rukarcheep, Suphot Wattanaphansak, Sumpun Thammacharoen, Chatsri Deachapunya, Sutthasinee Poonyachoti

**Affiliations:** 1 Faculty of Veterinary Science, Department of Physiology, Chulalongkorn University, Pathum Wan, Bangkok, Thailand; 2 Faculty of Veterinary Science, Department of Veterinary Medicine, Chulalongkorn University, Pathum Wan, Bangkok, Thailand; 3 Faculty of Medicine, Department of Physiology, Srinakharinwirot University, Wattana, Bangkok, Thailand; Children’s National Hospital, George Washington University, UNITED STATES

## Abstract

Modification of cellular and immunological events due to porcine reproductive and respiratory syndrome virus (PRRSV) infection is associated with pathogenesis in lungs. PRRSV also causes female reproductive dysfunction and persistent infection which can spread to fetus, stillbirth, and offspring. In this study, changes in cellular and innate immune responses to PRRSV type 1 or type 2 infection, including expression of PRRSV mediators, mRNA expression of Toll-like receptors (TLRs) and cytokine, and cytokine secretion, were examined in primary porcine glandular endometrial cells (PGE). Cell infectivity as observed by cytopathic effect (CPE), PRRSV nucleocapsid proteins, and viral nucleic acids was detected as early as two days post-infection (2 dpi) and persisted until 6 dpi. A higher percentage of CPE and PRRSV-positive cells were observed in type 2 infections. PRRSV mediator proteins, CD151, CD163, sialoadhesin (Sn), integrin and vimentin, were upregulated following type 1 and type 2 infection. *CD151*, *CD163* and *Sn* were upregulated by type 2. In both PRRSV types, mRNA expression of TLR1 and TLR6 was upregulated. However, *TLR3* was upregulated by type 1, but *TLR4* and *TLR8* mRNA and protein were downregulated by type 2 only. *Interleukin* (*IL*)-*1β*, *IL*-*6* and *tumor necrotic factor* (*TNF*)*-α* were upregulated by type 2, but *IL*-*8* was upregulated by type 1. Both PRRSV type 1 and 2 stimulated IL-6 but suppressed TNF-α secretion. In addition, IL-1β secretion was suppressed only by type 2. These findings reveal an important mechanism underlying the strategy of PRRSV infection in the endometrium and associated with the viral persistence.

## Introduction

Porcine reproductive and respiratory syndrome virus (PRRSV) disease is one of the most economic issue that affects the swine industry [1]. Massive reproductive disorders in sows causing perinatal losses and respiratory distress in piglets is the main impact of PRRSV outbreak [2]. Increased expenses associated with recirculation of PRRSV infection, i.e., confirming PRRSV status and treatment of secondary infections, have an indirect impact on the cost of production [1].

PRRSV is a positive single-stranded RNA virus belonging to the *Arteriviridae* family. PRRSV was first recognized in 1987 in the United States of America, and subsequently became a pandemic disease in North America, Europe, and Asia [3]. Two distinct strains, PRRSV type 1 (EU; Lelystad virus (LV) strain) and type 2 (US; VR-2332 virus strain), are identified and share approximately 60% genetic identity [3]. The genome of PRRSV is enclosed by nucleocapsid protein (N). The major envelope viral proteins, GP5 and M, form a heterodimer structure. The minor structural proteins which are GP2a, E, GP3 and GP4 (encoded from ORFs2-4), forms multimeric complex of GP2/GP3/GP4/E proteins. Different PRRSV genotypes have been suggested to relate with different severity and clinical outcomes [3]. PRRSV type 2 infection causes more severe respiratory distress than type 1 [4]; however, both genotypes cause reproductive failure at the same degree [5]. Besides, *in vivo* inoculation with PRRSV type 2 could not demonstrate the virulence of reproductive signs, i.e., viral load in fetus or maternal-fetal interface, numbers of embryonic death or PRRSV-positive litters, as compared to type 1 [6].

Among the identified virulent factors of each PRRSV genotype, non-structural glycoproteins (Nsp 3–8) and ORF5, encode structural protein GP5 [7]. The variation of these viral proteins between type 1 and type 2 infection appears to be the determinant of PRRSV virulence, pathogenicity, tissue susceptibility, and cell invasion ability. Recently, highly pathogenic PRRSV (HP-PRRSV), the virulent PRRSV NADC30 strains in the United States and the NADC30-like strains in China, have been emerged. However, HP-PRRSV appears to be the consequences of PRRSV evolution, i.e., variant of PRRSV type 2 [8,9].

PRRSV has a very specific cell tropism, which mainly infects cells in macrophage/monocyte lineages, in particular PAMs and other tissue macrophages [10,11]. PRRSV mediators are receptor proteins that determine cell tropism and play significant roles in PRRSV infection, including virus binding, internalization, uncoating, and replication [12]. At least five molecules have been described as PRRSV mediators, such as CD163, sialoadhesin (CD169; Sn), CD151, integrin, and vimentin [13,14].

For natural PRRSV infection in pigs, Sn and CD163 are identified as the classical PRRSV receptors and required for PRRSV infection. Sn mediates the virus binding and internalization, whereas CD163 mediates the uncoating and genome release [11]. During the initiation of PRRSV infection, binding of GP5-M heterodimer protein to Sn is subsequently internalized by endocytosis [15]. PRRSV genome is then released by interaction of its structural proteins GP2/GP3/GP4 with the other PRRSV receptor CD163 [16].

Furthermore, the established cell lines commonly used for *in vitro* PRRSV propagation, i.e., MA104 (an African monkey kidney cell line) or its derivatives MARC-145 and CL2621 cells, require the expression of the other mediators, such as vimentin and CD151 [17]. The expression of vimentin and CD151 is suggested to facilitate PRRSV infection by respectively interacting with PRRSV N protein and 3’ untranslated region (UTR) RNAs of the arteriviruses [18,19]. They also play an important role in virus replication through interactions with other cellular proteins [18]. In addition to the PRRSV specific receptors, adhesion molecules like integrin can facilitate many viral internalization and infection, including PRRSV infection in reproductive organs [20].

In reproductive organs, PRRSV can be transmitted via transplacental viral shedding leading to aborted fetus and/or weak born piglet [21]. It has been suggested that PRRSV replication can occur at the implantation site [22]. Previous study in primary porcine glandular endometrial cells (PGE) demonstrates a low expression level of PRRSV receptors, CD163 and Sn; however, PRRSV can induce CPE and damage PGE [23]. Moreover, high existence and release of PRRSV from PGE was gradually increased with time, suggesting the susceptibility or recirculation of PRRSV in the infected PGE [23]. Overexpression or modification of PRRSV receptor cDNA in PRRSV non-permissive cell lines can generate the infectious progeny virus from those cells [24]. This raised the possibility that PGE may express alternative PRRSV receptors, in which PRRSV can infect and process host cellular response. The modification of PRRSV receptor expression associated with the increased PRRSV susceptibility and replication remains to be investigated.

Innate immune response is primarily responsible for an immediate protection against pathogen invasion and subsequently activates the adaptive immune response. The innate immune response is mediated through host pattern recognition receptors (PRRs), i.e., toll-like receptors (TLRs). The TLRs 1–9 are expressed in immune cells and non-immune cells like endometrial epithelial cells [25]. Endosomal TLRs, TLR3, TLR7, TLR8, and TLR9 detect internalized viral nucleic acid and promote the production of anti-viral cytokines such as type I interferons (IFN), IFN-α and IFN-β [26]. Membranous TLRs, TLR2 and TLR4 generally recognize bacterial structural proteins but not the invading viral particles. TLR and cell signaling system mediate host-viral PRRSV interaction by inducing the production of pro-inflammatory cytokines, such as IL-6, IL-8 and TNF-α in MARC-145 and PAMs [27]. PRRSV infections are characterized by prolonged viremia and complications from immunosuppressive effects because of the upregulation of *IL*-*10* and *IL*-*1*β expression and reduction of *IFN*-*F067* in porcine polymorphonuclear cells [28,29]. Thus, the cellular modification of host immune system is relevant to both pathogenesis and host protection of PRRSV. The effect of PRRSV infection on the local innate immune responses of the reproductive system is associated with reproductive failure is still in question.

In the present study, we employed the PGE infected with PRRSV from our previous study [23] to determine an *in vitro* cellular response in association with cytopathic effect (CPE) and viral replication pertaining to the expression of aforementioned PRRSV mediators, TLRs and pro-inflammatory cytokines secretion following PRRSV type 1 or type 2 infection.

## Materials and methods

### Reagents and materials

Cell culture-grade reagents of Ringer’s solution, ethanol, isopropanol, H_2_O_2_ and methanol were purchased from Sigma Chemical Co., (St Louis, MO, USA). Dulbecco’s Modified Eagle’s Medium (DMEM), Dulbecco’s PBS, fetal bovine serum (FBS), collagenase type I, 0.05% trypsin-0.53 mM ethylenediaminetetraacetic acid (EDTA), kanamycin, penicillin-streptomycin and fungizone were purchased from Gibco BRL (Grand Island, NY, USA).

### Antibodies

Rabbit polyclonal antibody (pAb) against PRRSV envelop glycoprotein GP5 (Biorbyt Ltd., Cambridge, UK; dilution 1:100) was used to detect PRRSV protein. To localize PRRSV mediators, antibodies with the final dilution including rabbit pAb anti-human CD151 (Abcam, Waltham, MA, USA; 1:250), goat pAb-anti-porcine CD163 (Santa Cruz biotechnology, Dallas, TX, USA; 1:25), mouse monoclonal Ab (mAb)-anti-sialoadhesin (Bio-rad, Inc., Hercules, CA, USA; 1:250), goat pAb anti-integrin-α3 (Santa Cruz biotechnology, Dallas, TX, USA; 1:25) and mAb anti-vimentin (Santa Cruz biotechnology, Dallas, TX, USA; 1:250) were used for immunostaining. To assess all the expression of toll-like receptors, mAb anti-TLR were purchased from Santa Cruz biotechnology (Dallas, TX, USA). Other antibodies including mAb anti-β-actin, HRP-conjugated anti-mouse IgG, and mAb anti-goat IgG were purchased from Bio-rad (Hercules, CA, USA).

### Ethical statement for experimental procedures

All animal procedures performed in accordance with the Guide for the Care and Use of Laboratory Animals of the National Research Council of Thailand were reviewed and approved by The Institutional Animal Care and Use Committee (IACUC number 2231047) of Chulalongkorn University.

### PGE cell culture

PGE were isolated from Thai crossbred commercial 4–6 months old gilts provided by a government-qualified slaughterhouse in Bangkok, Thailand. According to the Animal Protection Law, pigs were slaughtered by stunning with subsequent carotid artery bleeding. Immediately after the carcass was opened along the longitudinal line, the uterine horn was taken and washed in Ca^2+^- and Mg^2+^-free PBS. The mucosal layer was stripped off, minced, and digested overnight with 0.2% collagenase. Endometrial glands were isolated from the digested tissues by filtration (40 µm pore size) followed by a gravitational sedimentation. The sedimented glands were collected and cultured in DMEM containing 5% FBS, 100 U/mL penicillin, 100 µg/mL streptomycin, 100 µg/mL kanamycin, 1% non-essential amino acids, and 10 µg/mL insulin at 37°C in 5% CO_2_. The culture media were refreshed every 2 days. The contamination of immune cells was removed, and the attached epithelial cells became confluent monolayers within 3–4 days. The remaining stromal cells were removed by adding 0.02% collagenase in serum-free medium for 24 h. After reaching 90% confluence, the cells were trypsinized and sub-cultured to an appropriate cell culture vessel for each experiment.

Following our previous study [30], PGE purity over 98% was determined by the immunocytochemistry staining of anti-pan cytokeratin antibody and transepithelial electrical resistance (TER) of greater than 400 Ω.cm^2^. PGE monolayers with TER of 400–800 Ω.cm^2^ which were considered as high tight junction integrity for studying ion transport were chosen for inoculation. The contamination of PGE with *Mycoplasma spp*., swine fever or PRRSV was assessed by a multiplex reverse transcription quantitative polymerase chain reaction (RT-qPCR) detection kit (Microplasma 16 s Ribosomal RNA Gene Genesig® Standard kit, [Primerdesign, Camberley, UK]; Virotype® CSFV RT-PCR kit, [QiagenIAGEN, Leipzig, Germany]) [23]. The mentioned pathogen-negative PGE was chosen for PRRSV inoculation.

### Preparation and quantification of PRRSV inoculum

PRRSV was isolated from the lungs of pigs with respiratory and reproductive illness and positive PRRSV sera at the Farm Animal Hospital (Faculty of Veterinary Science, Chulalongkorn University, Nakorn Pathom, Thailand). Upon arrival at the hospital, all the pigs were sedated with combination of xylazine (1.5 mg) and ketamine (11 mg/kg) intramuscularly followed by euthanasia with an overdose of intravenous pentobarbital sodium. To confirm and prepare PRRSV inoculum, 2.3 g of the infected lung tissues was minced, homogenized in 15 mL of cold FBS-free DMEM, and centrifuged at 10,000 × g and 4°C for 10 min following the previous protocol [31].

To assess the PRRSV genotypes, the supernatants were collected and filtered through a 0.2-µm syringe, diluted with FBS-free DMEM at a 1:1 ratio, and freshly proceeded to RT-qPCR using primers N26: GCCCTAATTGAATAGGTGAC; FT1: AGAAAAAGAAAAGTACAGCTCCGAT and N26/FT2.1: GTGAGCGGCAATTGTGTCTGTCG specific to ORF7 of type 1 / type 2, ORF 7 of type 1 and ORF 7 of type 2, respectively. According to our previous study [23], 1 µg of cDNA template was mixed with qPCR SYBR master mix in the presence of forward and reverse primers. All reactions were subjected to CFX96™ Real-Time PCR Detection System (Bio-rad, Hercules, CA, USA) using the following cycle: 95°C for 3 min to activate the reaction, followed by 40 cycles of amplification steps, including denaturation at 95°C for 20 s, annealing at 60℃ for 30 s and extension at 72℃ for 30 s, respectively. During the amplification, the numbers of cycle initially detecting the emission of SYBR green that incorporated into PCR product of each sample were recorded and reported as threshold cycle (*Ct*). Based on the previous study, PRRSV type 1 and type 2 inoculum at *Ct* = 35 were equivalent to 2.0x10^4^ PRRSV copies/µL [32]. The specificity of amplified products was confirmed using 1.5% agarose gel electrophoresis and melting curve analysis. The lungs of PRRSV-negative pigs were isolated and used for mock infection. No amplicons were produced in the mock control.

All PRRSV inoculum were quantitated using MARC-145 cells (ATCC American Type Culture Collection, VA, USA) cultivating in a 25-cm^2^ flask (Costar^®^, Corning, MA, USA) supplemented with DMEM containing 10% FBS. To quantitate the virus concentration as described previously [23], PRRSV inoculum at 1 mL of 10-fold serial dilutions (10^−1^ to 10^−6^) were incubated with the confluent MARC-145 cells at 37°C in 5% CO_2_ for 1 h. After viral inoculation, the cells were washed and replaced with the fresh media. CPEs in each MARC-145 cell culture well were observed microscopically at 2-, 4- and 6-days post-infection (dpi). Following the Reed-Muench method [33], the dilution that produced CPEs by 50% was considered the tissue culture infective dose 50% (TCID_50_)/mL. Based on the microtitration in MARC-145 cells and the Multiplex RT-qPCR assay, the approximate viral concentration of lung isolation in the present study were a 10^7^ TCID_50_/ml in all samples. PRRSV inoculum stock at the endpoint dilution of 10^5^ TCID_50_/mL to produce the pathological changes was used in this study [23]. The inoculum contaminated with *Mycoplasma spp*. and classical swine fever virus as confirmed by Multiplex RT-qPCR (Accessquick™, Promega) was excluded from the study.

### PRRSV inoculation

PGE (1x10^6^ cells/mL) were seeded into 24 mm membrane cell culture inserts (Transwell, MA, USA) or 25 cm^2^ flasks (Costar, MA, USA), and maintained in the culture medium for 7 days to become confluence. PGE were then allocated to Mock-, PRRSV type-1 or type-2 group (n = 5 pigs each group). According to our previous protocol [23], the PGE monolayer in 24 mm culture inserts or 25 cm^2^ flasks was respectively inoculated with 1 mL or 5 mL of PRRSV 10^5^ TCID_50_/mL inoculum in 5% CO_2_ at 37°C for 1 h. Inoculation with solution extracted from PRRSV-negative lungs was used for the mock group. After 1 h of viral adsorption, the cells were washed and replaced with fresh medium for 2–6 days. Each inoculation was performed in duplicate.

### Immunohistochemistry, detection, and quantitation of PRRSV infection and mediators

All PRRSV-infected PGE were confirmed by the presence of CPE at 2, 4 and 6 dpi. All CPEs were observed and measured in filter-grown PGE under light microscope with digital camera (BX50F and UC50, Olympus, Tokyo, Japan). The total area of CPE was normalized with the membrane filter area and reported as a percentage. To detect and quantitate existent PRRSV, PRRSV isolated from PGE and culture medium were assessed by RT-qPCR with the inoculation to MARC-145 cells following the Reed-Muench method [33].

Following the detection of CPE in PRRSV-infected PGE, immunostaining with primary antibodies (Ab) to recognize viral protein PRRSV-GP5, and PRRSV mediators CD151, CD163, sialoadhesin (Sn), integrin, and vimentin was performed. Following our previous protocol [23], filter-grown PGE were fixed with 4% paraformaldehyde in PBS, pH 7.4 for 10 min at 25°C. Fixed PGEs were treated with the non-specific blocking solution, 10% H_2_O_2_ in methanol, followed by 4% goat serum in PBS for 4 h. The treated samples were incubated with primary antibodies at 4°C overnight followed by universal HRP-conjugated secondary antibodies (Vectastain^®^ Elite ABC-HRP kit, Vector Laboratories, Inc., Burlingame, CA, USA). Immunoreactivity was developed by incubating membrane filter with DAB (3,3-diaminobenzidine tetrahydrochloride) substrate (Sigma Aldrich, MO, USA) and counter-stained with hematoxylin (Invitrogen, Waltham, MA, USA). Immunoreactive area was visualized under light microscope connected to a digital camera (BX50F and UC50, Olympus, Tokyo, Japan). All positive cells were captured by the digital images at the magnification of 20x and analyzed by ImageJ software (National Institutes of Health, Bethesda, MD, USA). The area in pixel numbers of immunoreactive cells (dark-brownish staining) was measured and calculated as the percentage of the total area of PGE, which comprised immunoreactive and non-immunoreactive cells per fields. The means of all percentages of positive cells in each group were compared.

### Total RNA isolation and reverse transcription

Total RNA was collected from PGE (uninfected, mock, type 1, and type 2 groups) grown in T25 flask using TRIzol® reagent (Invitrogen, Waltham, MA, USA). The concentration of total RNA was measured for optical density at 260 nm (OD_260_) using Nanodrop (NanoDrop 2000, Thermo Fisher Scientific, Waltham, MA USA). The purity of RNA was acceptable if the OD_260_/OD_280_ ratio was at 1.8–2.0. Reverse transcription was done by using iScript® Select cDNA synthesis Kit (Bio-Rad Laboratories, Hercules, CA, USA). Briefly, a 20 µl of reaction mixture consisting of total RNA 3 µg, oligo dT primer and iScript reaction was prepared. First-strand DNA was synthesized in thermocycler (Biometra GmbH, Göttingen, Germany) using the following cycle: 25°C for 3 min, 46°C for 20 min, and 95°C for 1 min. cDNA concentration was measured using NanoDrop and product stored at -20°C until performing real-time PCR.

### Real-time PCR

The mRNA expression of PRRSV mediators, TLRs, and cytokines was carried out using a GeneOn SYBR green based qPCR kit (GeneOn, Deutschland, Germany) following the previous protocol [23,34]. Briefly, a 20 µl of PCR reaction containing 1 µg of cDNA template, 2x qPCR SYBR master mix, and a pair of forward and reverse primers for each gene (Table 1) was prepared. Forty cycles of reaction at 95°C, 60°C, and 72°C for 20, 30, and 30 s, respectively, were carried out in a DNA Thermal Cycler (CFX96^TM^, Bio-rad, Hercules, CA, USA). The amplicons were evaluated for the specificity of product by running 1.5% agarose gel electrophoresis and analyzing melting curve. The relative mRNA expression of interested genes was determined by normalization with GAPDH mRNA expression and reported as fold change compared to those of uninfected PGE using 2^*-∆∆Ct*^ calculation [35].

**Table 1:** Porcine sequence of primer sets used in this study.

Gene	Primer sequences (5’→ 3’)	Accession number	Expected size (bp)
*CD151*	Fwd: TGTGTGCAGGTGTTCGGCAT Rev: TCAGCGCATCCTGAGAAGCT	NM_001243865.1	125
*CD163*	Fwd: AATTCCAGTGTGAGGGGCAC Rev: AGCGGATTTGTGTGTATCTTGAG	HM991330.1	122
*Integrin*	Fwd: GACCAGGTGACCCGTTTCAA Rev: TCCAGCCAATCTTCTCGTCAC	NM_214002.1	124
*Sn*	Fwd: TCGATGCCCAGGCTATGAGA Rev: CAGGTGCGTGGTCCATACAA	NM_214346.1	113
*Vimentin*	Fwd: TCCAAGTTTGCCGACCTCTC Rev: GACTCGTTGGTCCCCTTGAG	XM_005668107.1	140
*TLR1*	Fwd: CACAGAGTCTGCACATTGTTTATCC Rev: GATTTACTGCGGTGCTGACTGA	NM_001031775.1	74
*TLR2*	Fwd: GTGCTTTCCGAGAACTTTGT Rev: GCAGAATGAGGATGGCG	KF460452.1	134
*TLR3*	Fwd: TCCAACTAACAAACCAGGC Rev: ACATCCTTCCACCATCT	NM_001097444.1	81
*TLR4*	Fwd: AAGGTTATTGTCGTGGTGT Rev: CTGCTGAGAAGGCGATAC	NM_001293316.1	114
*TLR5*	Fwd: TTGCATCCAGATGCTTTTCA Rev: TTCAACTTCCCAAATGAAGGA	XM_012506471.1	122
*TLR6*	Fwd: TCACCTCTCTGACATCAGCTTTCT Rev: TGATATCAAGGCACTGCATCCT	NM_213660.1	116
*TLR7*	Fwd: GGACCATCTGGTAGAGATCGATTT Rev: TTCTGGTGCACAGGTTGTCTTT	NM_001097434.1	101
*TLR8*	Fwd: CCGCACTTCGCTATCTAAAC Rev: GAAAGCAGCGTCATCATCAA	NM_214187.1	72
*TLR9*	Fwd: AGATGTTTGCTCGCCT Rev: GGACACTCGGCTATGGA	KC860785.1	126
*TLR10*	Fwd: CTACCAGGTATCCTGCACTGAAAG Rev: GGCAACATTTACGCCTATCCTT	NM_001030534.1	110
*IL-1β*	Fwd: AACGTGCAGTCTATGGAGT Rev: GAACACCACTTCTCTCTTCA	M86725	82
*IL-6*	Fwd: AGATGCCAAAGGTGATGCCA Rev: ACAAGACCGGTGGTGATTCTCA	NM_214399	195
*IL-8*	Fwd: TTTCTGCAGCTCCTCTGTGAGG Rev: CTGCTGTTGTTGTTGCTTCTC	M99367 M86923	129
*IFN-γ*	Fwd: GTTTTTCTGGCTCTTACTGC Rev: CCTCCGCTTTCTTAGGTTAG	X53085	166
*TNF-*α	Fwd: ATCGGCCCCCAGAAGGAAGAG Rev: GATGGCAGAGAGGAGGTTGAC	M29079 X54859	140
*GAPDH*	Fwd: GGACCAGGTTGTGTCCTGTGA Rev: TCCACCACCCTGTTGCTGTAG	NM_001206359.1	144

### Semiquantitative Western blot analysis

PGE proteins were harvested using lysis buffer containing 50 mM tris, 150 mM NaCl, 1mM EGTA, 1mM PMSF, 1% NP-40, 6.02 mM sodium deoxycholate, 0.01 mg/ml aprotinin, 1 mM NaF and a cocktail protease inhibitor. Cell lysate was centrifuged at 12000 rpm for 15 min at 4°C. The supernatant was collected and measured for a protein concentration using the BCA^TM^ protein assay (Thermo Fisher Scientific, MA, USA). The protein sample was diluted with Laemmli buffer containing β-mercaptoethanol (Bio-rad, Hercules, CA, USA) and incubated at 65°C for 5 min. The obtained denatured protein (30 μg) was separated by 10% sodium dodecyl sulphate-polyacrylamide gel electrophoresis (SDS-PAGE) and blotted to a PVDF membrane (Millipore®, St Louis, MO, USA). After blocking non-specific protein with 2% bovine serum albumin, the blotted membrane was probed with primary antibodies at 4°C for 12 h and further stained with HRP-conjugated secondary antibodies for 1 h at room temperature. Dilution of all antibodies was used following the manufacturer’s instruction. An ECL substrate (Santacruz Biotechnology, Dallas, TX, USA) was used to develop immunoreactive bands, which were visualized by exposure to an X-ray film (GE healthcare, Bloomington, IL, USA). The protein expression of TLRs (n = 5 pigs each group) was analyzed by Scion image software and normalized to β-actin. The results were represented as relative fold changes of protein expression over those of mock group.

### Measurement of cytokine secretion

Media collected from the apical and basolateral compartment of filter-grown PGE at 0, 2, 4 and 6 dpi were determined for cytokine secretion, which consisted of chemokine (C-C motif) ligand 2 (CCL-2), IL-1β, IL-6, IL-8, IL-10, TNF-α, IFN-α and IFN-γ by enzyme-linked immunosorbent assay (ELISA) using commercial kits (R&D System, Minneapolis, MN, USA). The ELISA processes were performed according to the instruction of commercial kits (n = 5 pigs for each group). Cytokine concentration was measured at OD_450_, by a microplate reader (Biotek, Winooski, VT, USA), in which a background OD_620_ was subtracted from all data. The concentration was calculated from a standard curve. The accumulated concentrations of each cytokine from 0–6 dpi were normalized by dividing with those of uninfected cells and displayed as fold changes of uninfected cells.

### Statistical analysis

All PGE data were obtained from 5 pigs per group and shown as the mean ± SEM. Statistical analysis was done by GraphPad Prism 9.0 (GraphPad software Inc., San Diego, CA, USA). The percentages of CPE and PRRSV positive cells among different dpi were analyzed by two-way ANOVA, and the mRNA expression of interested genes, protein expression, and cytokine secretion were analyzed by one-way ANOVA. The multiple comparison with the uninfected group was performed by Bonferroni’s post hoc test. A *p* value of less than 0.05 was considered as significant differences.

## Results

### PRRSV type 2 induced CPE and viral existent in PGE

**Fig 1:**
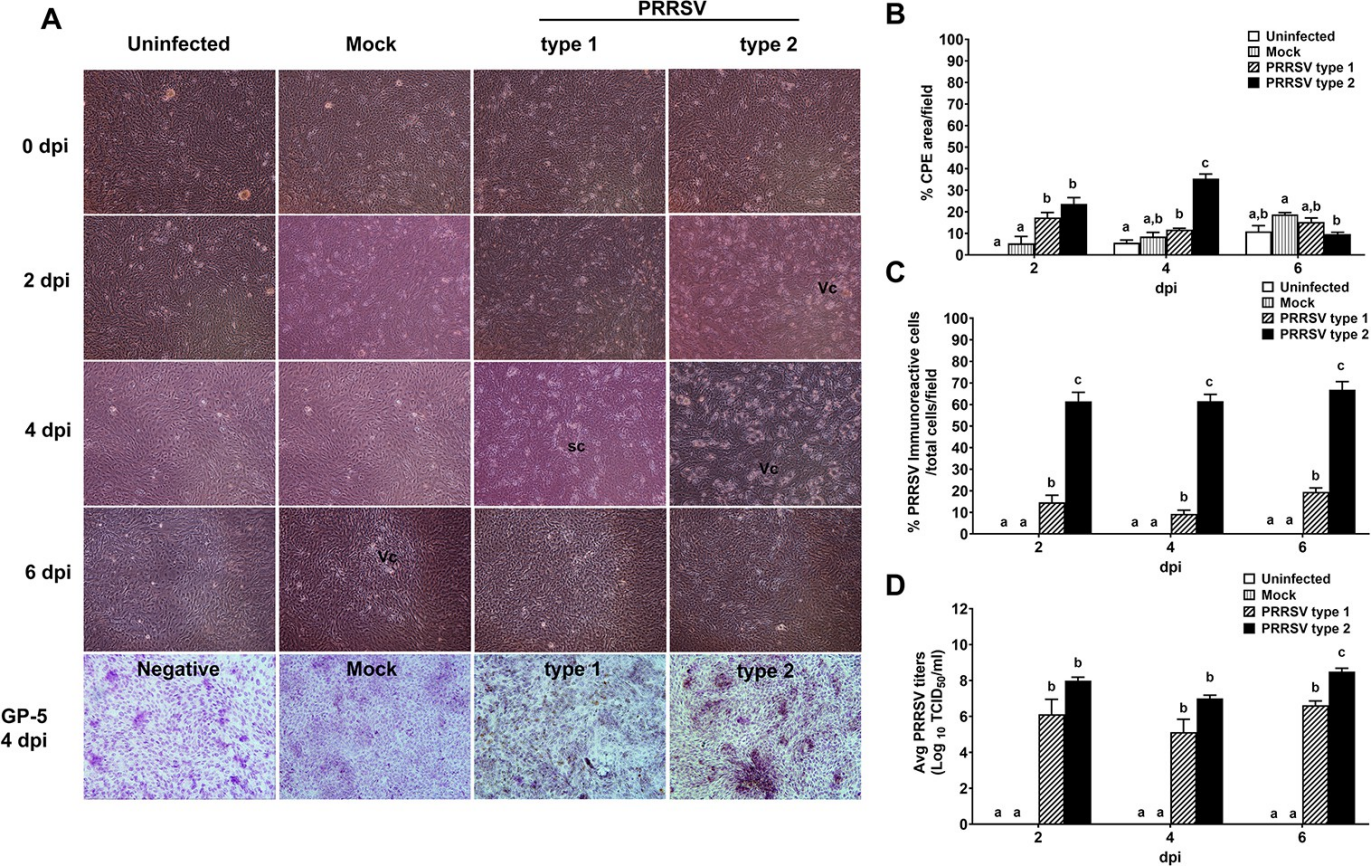
Type 2 PRRSV-infected PGE demonstrated high cytopathic effects (CPE), PRRSV-positive cells and viral titers. PGE monolayers infected with mock, type 1, or type 2 at 10^5^ TCID_50_/mL were observed for CPE, and PRRSV positive cells were investigated by immunocytochemistry using antibody against PRRSV envelop glycoprotein GP5. A) Representative images of cellular morphological changes of uninfected, mock- and PRRSV-infected PGE at 2-, 4- and 6-days post-infection (dpi). The scale bar = 500 µm. B) Percentage of CPE at 2, 4 and 6 dpi. The CPE area was increased at 2 and 4 dpi, but not 6 dpi, by type 1 and 2 infection. C) Percentage of PRRSV positive PGE at 2, 4 and 6 dpi. At all dpi, the PRRSV positive cells was increased by 10–20% following type 1 and up to 60% following type 2 infection. D) PRRSV titers in PGE at 2, 4 and 6 dpi. The highest titers of viral (TCID_50_/mL) were detected in type 2 infected PGE at 6 dpi. Bar graphs represent mean ± SEM (n = 5 pigs). Difference between PRRSV type and date of infection was analyzed by *ANOVA* and Bonferroni’s post hoc test. Bar graph with different letters indicates significantly different (*p* < 0.05).

As shown in Fig 1, PGE infected with PRRSV type 1 or type 2 showed CPE, including syncytial formation (Sc; Fig 1A) and vacuolization (Vc; Fig 1A) as early as 2 dpi. The CPE area at 2 dpi were significantly extended to 20–30% in both type 1 and type 2 inoculated PGE as compared to mock and uninfected PGE (Fig 1B). A greater extent of CPE area (40%) was found in type 2 inoculated PGE at 4 dpi, but it was recovered at 6 dpi (Fig 1B). By contrast, the CPE area produced by type 1 were not significantly different from that by mock and uninfected groups at 6 dpi (Fig 1B). Corresponding to CPE formation, immunoreactivity of PRRSV envelop protein (GP5; Fig 1C) was early observed at 2 dpi and remained up to 6 dpi. Type 2 inoculation increased the PGE immunoreactive cells about 3–4 times higher than type 1 inoculation (Fig 1C, *p* < 0.05).

The PRRSV existing in type 2-inoculated PGE was 10^9^ TCID_50_/mL which were a ten-fold higher than type 1 at 6 dpi (Fig 1D; *p* < 0.05). However, the PRRSV titers were equivalent in both type 1 and type 2 infected PGE at 2 and 4 dpi (Fig 1D; *p >* 0.05).

### PRRSV type 2 upregulated *CD151*, *CD163* and *Sn* mRNA expression

**Fig 2:**
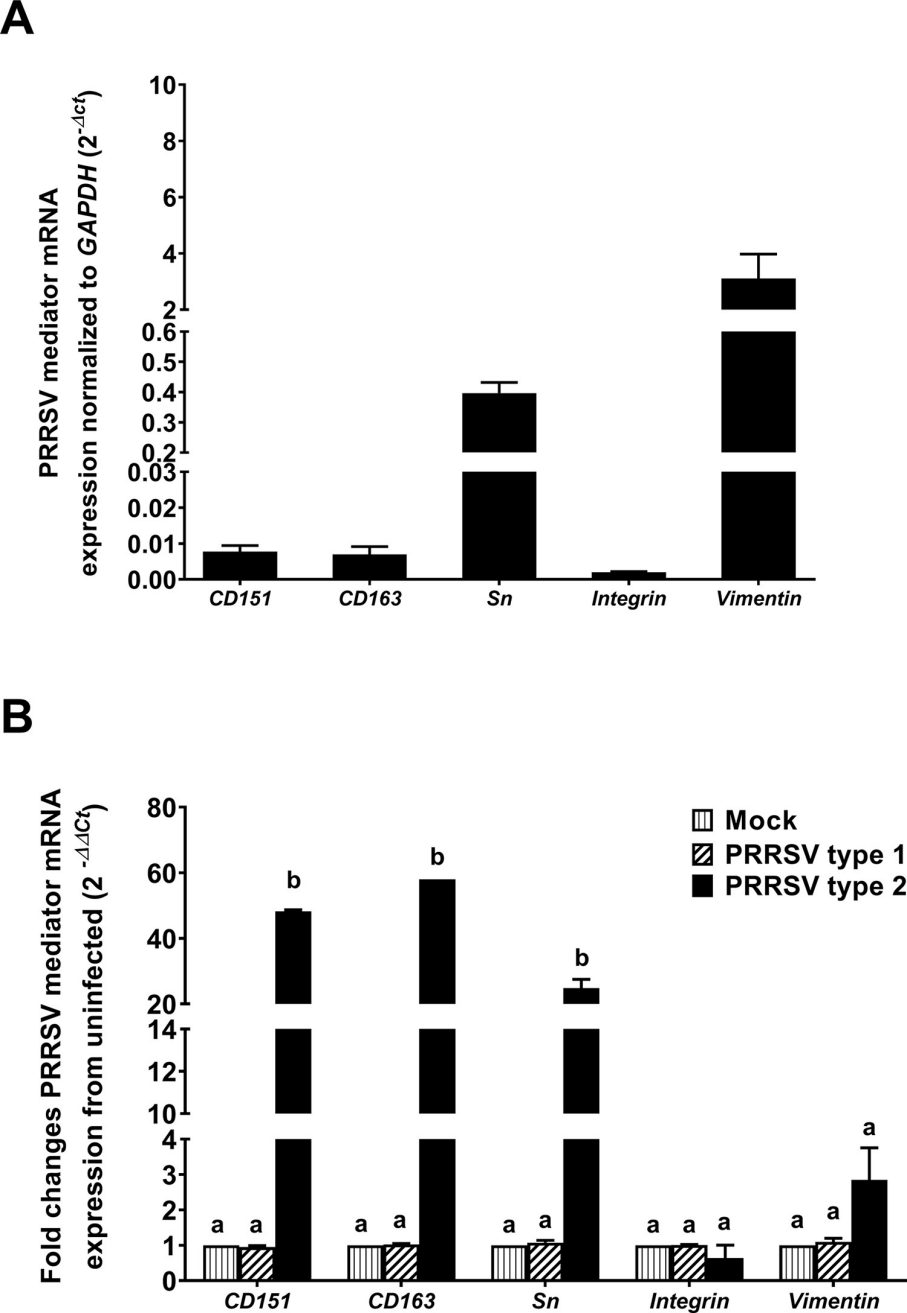
Type 2 PRRSV-infected PGE upregulated *CD151* and *CD163*. Total RNA was isolated from PGE infected with mock, type 1, or type 2 at 10^5^ TCID_50_/mL at 4 dpi to determine mRNA expression of PRRSV mediators using real-time PCR. A) The expression of PRRSV mediators in uninfected PGE normalized to house-keeping gene *GAPDH* as calculated by *2*^*-∆Ct*^. B) Relative mRNA expression of PRRSV mediators compared to uninfected cells using the *2*^*-∆∆Ct*^ calculation. *CD151*, *CD163* and *Sn* were upregulated by type 2 infection. Bar graphs represent mean ± SEM (n = 5 pigs). Bar graph with different letters indicates significantly different (*p* < 0.05) as analyzed by *ANOVA* and Bonferroni’s post hoc test.

The mRNA expression of PRRSV mediators, *CD151*, *CD163*, *Sn*, *integrin*, and *vimentin*, was determined at 4 dpi to examine whether they might be a target involved in PRRSV infection and existence. The normal uninfected PGE expressed a relatively higher level of *vimentin* (3-fold) and low level of *CD151*, *CD163*, *Sn* and *integrin* (0.5-fold) as normalized to *GAPDH* (Fig 2A). At 4 dpi, only type 2 infection was found to upregulate the expression of *CD151*, *CD163* and *Sn* by 4-60-fold (*p* < 0.05, Fig 2B) with no effects on *integrin* or *vimentin*. However, both mock and type 1 infection could not produce any effects on the mRNA expression of PRRSV mediators tested (*p* > 0.05; Fig 2B).

### PRRSV type 1 and type 2 upregulated cellular expression of PRRSV mediators

**Fig 3:**
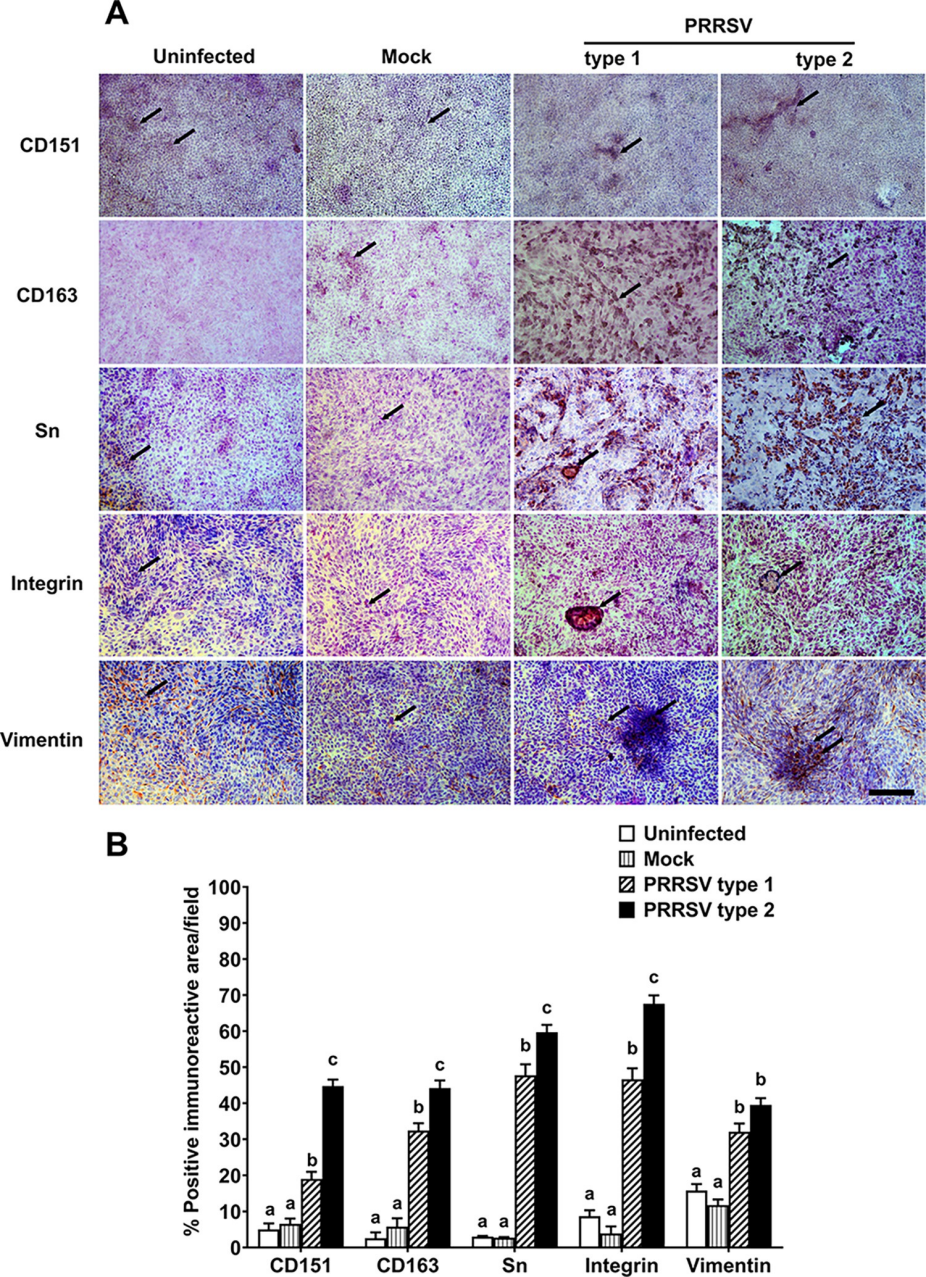
PRRSV upregulated cellular expression of PRRSV mediators. PGE monolayers infected with mock, type 1, or type 2 at 10^5^ TCID_50_/mL were fixed with 4% paraformaldehyde to observe protein expression of PRRSV mediators at 4 dpi using immunocytochemistry. A) Representative images of PRRSV mediator protein immunoreactivity (arrow) are demonstrated in uninfected, mock- and PRRSV-infected PGE. The scale bar = 500 µm. B) The percentage of positive PRRSV mediator immunoreactive area, as normalized to the total area of confluent cells, was upregulated by type1 and type 2 infection (*p* < 0.05). Bar graphs represent mean ± SEM (3 independent areas, n = 5 pigs). Bar graph with different letters indicates significantly different (*p* < 0.05) as analyzed by ANOVA and Bonferroni’s post hoc test.

Apart from mRNA expression, cellular localization of PRRSV mediator proteins at 4 dpi was further evaluated using immunohistochemistry. In uninfected PGE, at 0 dpi, the immunoreactivity of CD151, CD163, Sn and integrin was rarely detected, whereas that of vimentin was demonstrated about 10% (Fig 3A). The immunoreactive CD151, CD163, Sn or integrin was distributed in the cytoplasm of mock or PRRSV infected PGE (Fig 3A). In contrast, the vimentin immunoreactivity which had fiber-like characteristics was observed in the uninfected and mock PGE. Analysis of expression area of PRRSV mediators was not difference between the uninfected and mock PGE (*p* > 0.05; Fig 3A and 3B). In contrast to PRRSV receptor mRNA expression results in Fig 2B, both type 1 and type 2 markedly upregulated the expression of all PRRSV mediators. In this result, the increases in CD151, CD163, Sn, and integrin expression induced by type 2 was higher than type 1 (*p* < 0.05; Fig 3B).

### PRRSV type 1 and type 2 regulated different types of TLR expression

**Fig 4:**
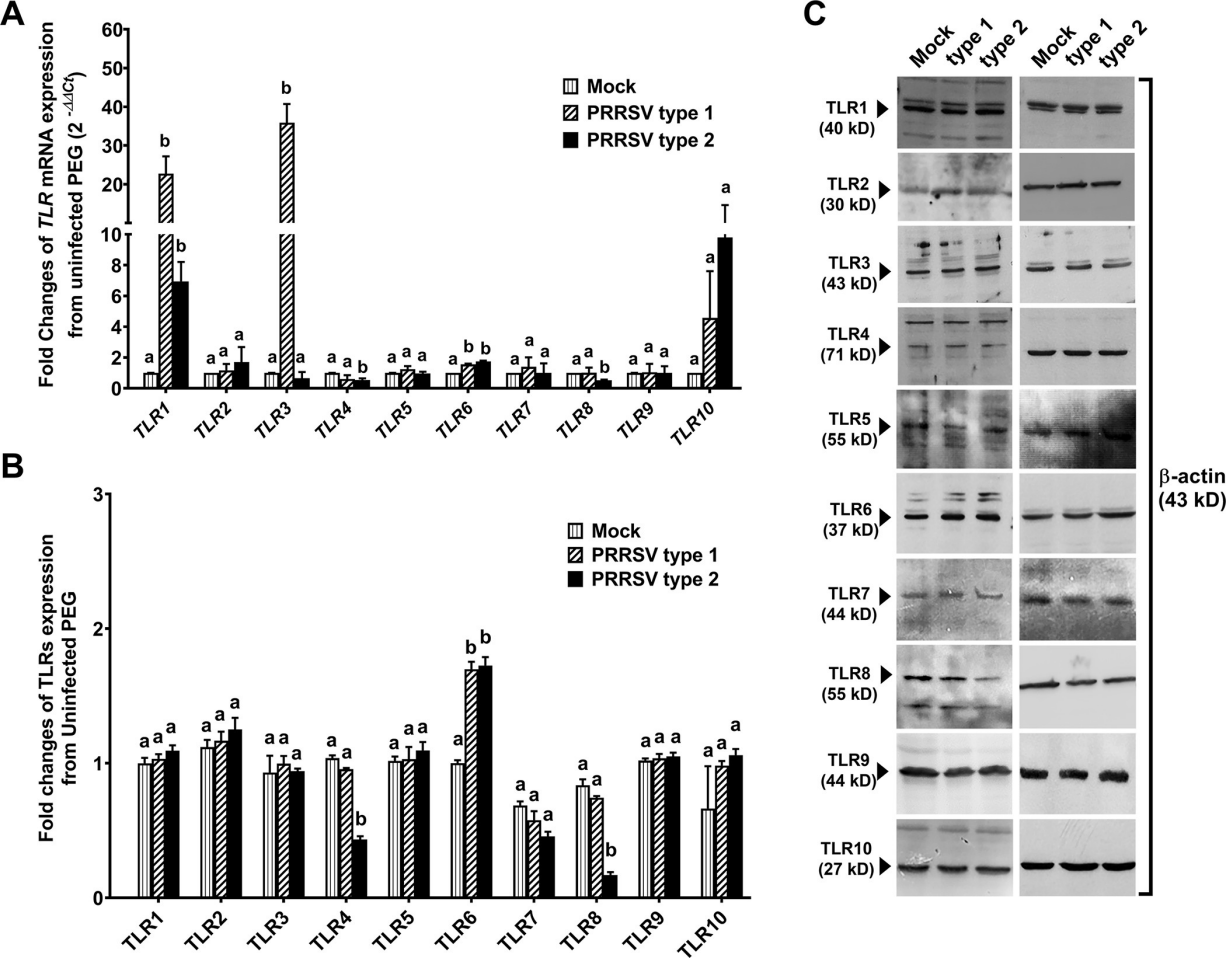
PRRSV-infected PGE altered TLR expression. Total RNA and protein were extracted from PGE infected with mock, type 1, or type 2 at 10^5^ TCID_50_/mL at 4 dpi to determine mRNA expression using reverse transcription and real-time PCR and protein using semi-quantitative Western blot analysis. A) Fold change of *TLRs* expression in PRRSV-infected PGE compared to uninfected cells using the *2*^*-∆∆Ct*^ calculation. Type 1 upregulated *TLR1*, *TLR3* and *TLR6*. Type 2 upregulated *TLR1* and *TLR6* but downregulated *TLR4* and *TLR8*. B) Fold change of all TLRs (1–10) protein expressions normalized to β-actin in PRRSV-infected PGE compared to uninfected cells. C) Representative band intensity of TLRs and β-actin was measured by ImageJ software. TLR6 protein expression was upregulated by type 1 and type 2. Only type 2 downregulated TLR4 and TLR8 expression. The scale bar = 500 µm. Bar graphs represent mean ± SEM (n = 5 pigs). Bar graph with different letters indicates significantly different (*p* < 0.05) as analyzed by ANOVA and Bonferroni’s post hoc test.

Changes in mRNA and protein expressions of TLRs 1–10 by PGE were also determined following 4 dpi. RT-PCR results of *TLR1*-*10* gene expression in Fig 4A showed that PRSSV type 1 and type 2 infection upregulated *TLR1* and *TLR6*, while only type 1 upregulated *TLR3* expression (*p* < 0.05; Fig 4A). In contrast, *TLR4* and *TLR8* was downregulated in type 2 infected group (*p* < 0.05; Fig 4A). In addition, PRRSV induced changes in the protein expression of TLR1-10 was mostly correlated with TLRs mRNA expression with the exception of TLR1 and TLR3 (Fig 4B and 4C). Of particular interest, both PRRSV type 1 and type 2 upregulated TLR6 by 2 fold from uninfected group (*p* < 0.05; Fig 4B), whereas type 2 downregulated TLR4 and TLR8 > 2 fold from uninfected group (*p* < 0.05; Fig 4B).

### PRRSV type 1 and type 2 upregulated IL-6 and downregulated TNF-α

**Fig 5:**
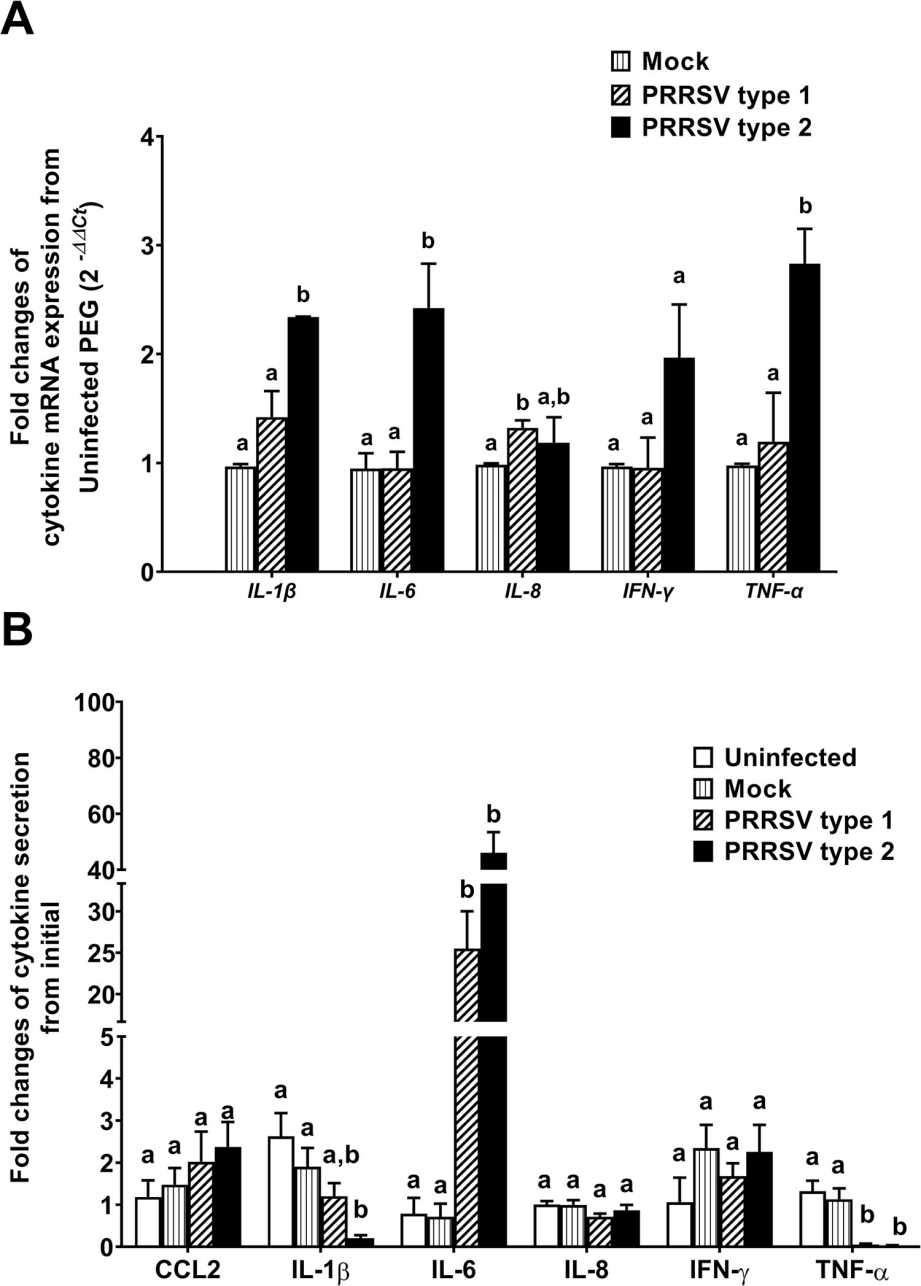
PRRSV-infected PGE upregulated IL-6 but downregulated TNF-F061 secretion. Total RNA and protein were extracted from PGE infected with mock, type 1, or type 2 at 10^5^ TCID_50_/mL at 4 dpi to observe mRNA expression of cytokines using real-time PCR. Media were collected at 2, 4 and 6 dpi to evaluate accumulated cytokine secretion using ELISA. A) Relative expression of *IL-6*, *IL-8*, *IFN-F067* and *TNF-α* normalized to *GAPDH* was compared to uninfected cells using the *2*^*-∆∆Ct*^ calculation. Type 1 upregulated *IL-8* whereas type 2 PRRSV upregulated *IL-1β*, *IL-6* and *TNF-α*. B) Relative accumulated cytokine secretion from uninfected cells. IL-6 and TNF-*α* secretion was respectively upregulated and downregulated by both PRRSV 1 and 2. IL-1β secretion was downregulated only by type 2. Bar graphs represent mean ± SEM (n = 5 pigs). Bar graph with different letters indicates significantly different (p < 0.05) as analyzed by ANOVA and Bonferroni’s post hoc test.

The mRNA expression of cytokines at 4 dpi and accumulated cytokine secretion at 6 dpi were determined following PRRSV inoculation in PGE. The mRNA expression of cytokines was not difference between uninfected and mock infected groups (*p* > 0.05, Fig 5A). *IL-8* was upregulated by type 1, and *IL-1β*, *IL-6* and *TNF-α* were upregulated by type 2 infection (*p* < 0.05; Fig 5A).

In PGE, many cytokines were secreted into media collected from the apical and basolateral compartment of filter-grown PGE, except IL-10 and IFN-α. Both PRRSV type 1 and 2 stimulated IL-6 but suppressed TNF-α secretion (*p* < 0.05; Fig 5B). The accumulated secretion of IL-1β was additionally suppressed after infection with type 2 (*p* < 0.05; Fig 5B). CCL2, IL-8 and IFN-γ secretions were not different among groups (*p* > 0.05; Fig 5B). Moreover, there was no difference in cytokine secretion between mock and uninfected groups (*p* > 0.05; Fig 5B).

## Discussion

Besides alveolar macrophages, the natural cell tropism of PRRSV, PGE has been recently reported as an alternative target of PRRSV, which was preferentially infected via the apical side of PGE monolayer [23]. In the present study, we examined the outcome of PRRSV infection in PGE from the beginning to the end stage (2–6 dpi) associated with their cellular response. Exposure of PGE to the PRRSV-isolated inoculum for 1 h demonstrated microscopic changes of CPE (Fig 1A and 1B), PRRSV protein (Fig 1C) and genomes (Fig 1D), which were observed as early as 2 dpi and remained up to 6 dpi. The PRRSV-induced CPE was demonstrated as disseminated vacuolization reflecting focal degeneration and cell death maximum at 4 dpi (Fig 1A). However, the degenerative vacuole was recovered showing less CPE with the persistent PRRSV (Fig 1B) at 6 dpi in all PRRSV infected PGE. Thereby, the compromising factors for PRRSV surviving, i.e., suppression of pro-inflammatory cytokines synthesis and release by PRRSV at 4 dpi was intrigued.

In the current study, PRRSV type 2 infected PGE displayed more CPE and higher percentage of PRRSV positive cells than type 1. Thus, more cellular destruction and PRRSV existence caused by type 2 infection indicated the higher virulence to PGE than that by type 1. This finding was consistent with natural infection, which PRRSV type 2 revealed more severity of respiratory distress than type 1 [3]. However, the natural infection or *in vivo* intranasal inoculation with type 1 or type 2 could not demonstrate the different virulence in reproductive signs, i.e., viral load in fetus or maternal-fetal interface, the number of embryonic death or PRRSV-positive litters [36]. In our previous studies, apical route of PRRSV infection alters the permeability and viability of PGE [23,34]. However, the basolateral route of PRRSV infection at the endometrium occurred following viremia also causes reproductive disorders [37]. Therefore, the different route of PRRSV infection which appears to produce severity in reproductive tissue dysfunction should be taken into consideration and confirmed by further study.

Our study is the first report to indicate the upregulated cellular expression of PRRSV mediators in response to PRRSV infection in porcine endometrial cells. PGE infected with PRRSV type 2 induced higher degree of upregulated *CD151*, *CD163* and *Sn* mRNA associated with the high titers of PRRSV persistence and release from infected cells. The increased PRRSV mediators in PGE reflects their substantial role in the sensitivity and persistence to the consecutive PRRSV infection.

In the present study, the classical PRRSV mediator CD163 was not expressed, but Sn was expressed at a low level in PGE cells. Sn (CD169 or Sialec-1) is a type I transmembrane glycoprotein consisting of extracellular Ig-like domains and a short cytoplasmic tail which can bind to sialic acid on GP5 protein allowing PRRSV to internalization [38]. After internalization, CD163 is essential for uncoating and releasing of the PRRSV in macrophage [39]. In our model, a common phenotype of non-infected PGE is recently characterized as Sn^+^/CD163^-^; however, these cells could be infected by PRRSV after inoculation.

Perhaps, the putative mediator CD151, integrin and vimentin expressed in non-infected PGE may be associated with PRRSV viral entry and host response during the early stage of infection. Although the mechanism of the putative receptors mediating pathogenesis of PRRSV infection were not examined in this study, the significance of these mediators on PRRSV infections were taken into account.

CD151 is a 29-kDa TM glycoprotein protein, belonging to the tetraspanin superfamily [12]. CD151 has been commonly expressed in PRRSV permissive cell lines including MA-104, and MARC-145, as well as in normal and cancer cell lines of human endometrium (RL95-2 and HEC-1-A) [12,40]. In addition to an important role in PRRSV replication [18,19], CD151 interacts with integrin to promote outgrowth in human embryonic carcinoma cell line NT2N [41]. Therefore, the upregulated CD151 at 4 dpi may explain the regeneration of PGE at 6 dpi in the present study, as well as stimulation of cell proliferation of PRRSV-infected PGE in our previous study [34]. The recovered PGE and overexpressed CD151 may assist PRRSV remaining or replication in host cells.

Integrin is a transmembrane glycoprotein expressed on many mammalian cell types. Functions of integrin are cellular adhesion, cell migration and signal transduction [42]. Integrin mediates viral internalization for rotavirus, foot-and-mouth-disease virus, simian virus 40 and HIV-1 integrin for infection [20]. It is likely that integrin may be a key mediator for PRRSV entry in the endometrium. However, the expression of integrin in PGE cells has been modulated by PRRSV. According to its function on cell migration and adhesion, the induction of integrin expression by PRRSV on 4 dpi may be relevant to the recovery of PGE at 6 dpi in our study (Fig 1B).

Vimentin, also known as fibroblast intermediate filament, is the major intermediate filament found in non-muscle cells and cancer cells [12]. However, vimentin has been identified as putative PRRSV receptor on MARC-145. [18]. It was suggested to interact with other cytoskeletal molecules for facilitating PRRSV replication [12]. During PRRSV infection, vimentin expression was up-regulated in PAMs and MARC-145 [43]. In PGE, PRRSV infection did not change *vimentin* mRNA expression although protein expression was abundantly localized at the degenerative cells (Fig 3A). It is possible that PRRSV infection modulated vimentin localization but not de novo synthesis. The modification of vimentin in infected PGE at 4 dpi was relevant to rearrangement or recovery of cellular structure and integrity in endometrial cells at 6 dpi.

It seems that the putative PRRSV receptors, CD151, integrin and vimentin, primarily expressed on PGE mediates viral infection and cellular responses at the early infection. Internalized PRRSV subsequently upregulated all types of PRRSV mediators, both classical and putative mediators. The upregulation of CD163 and Sn expressions may facilitate PRRSV re-infection, whereas the upregulation of CD151, integrin and vimentin help new arrangement of PGE for PRRSV replication and persistence.

There has been reported that many cellular responses including PRRSV receptors, i.e., CD163 and Sn are regulated by pro-inflammatory and anti-inflammatory mediators such as lipopolysaccharide, IFN-γ, TNF-α, IL-6 and IL-10 [44]. Basically, cytokines are released from immune cells in response to pathogens through TLR signaling pathway [26]. Several TLRs, TLR3 and TLR9 can recognize viral nucleic acids or viral protein produced by PRRSV leading to release many cytokines, such as IL-6, TNF-α and IFN-γ at the early stage of infection [45].

Upregulated TLR3, TLR7 and TLR8 expressions were correlated to PRRSV virulence and clinical signs in highly pathogenic PRRSV-infected pigs [46]. Differential regulation on *TLR1-10* expression was also observed following type 1 or type 2 PRRSV infection in PGE. Type 1 infection upregulated *TLRs*, *TLR1*, *TLR3* and *TLR6* mRNA expression but not the protein expression at 4 dpi (Fig 4). It is possible that the increase of those TLRs protein may be exhibited later.

Remarkably, highly pathogenic PRRSV was a stronger inducer of TLR3, 7 and 8 expressions [46]. Excessive expression of IL-6 followed by the increased expressions of pro-inflammatory cytokines and chemokines, including IL-1β, IL-12, IL-8, and TNF-α was relevant to the severe lung injury and damages of lymphoid organs [8]. Unlikely, PRRSV type 2 induced upregulation of TLR6, and downregulation of TLR4 and TLR8 mRNA and protein coincided with the decreased IL-1β and TNF-α secretion in type 2 infected cells at 4 dpi. These results may be associated with the persistence of PRRSV in PGE at 6 dpi.

In general, pro-inflammatory cytokines IL-1β and TNF-α are released by host to encounter invasive pathogens including viruses. The downregulated TNF-α synthesis and secretion by PRRSV infection in PGE were consistent to the study of PAMs infected with PRRSV type 2 [47]. Incubation with recombinant TNF-α was reported to reduce existent PRRSV [47]. In PRRSV-infected PGE, particularly type 2, the increased viral existence at 6 dpi (Fig 1D) was concurrent to the decreased IL-1β and TNF-α.

The suppressed TNF-α induced by PRRSV has been indicated to carry out by Nsp1 by inhibiting the activation of NF-κB and Sp1 transcription factors on the promoter region of *TNF-α* [48], whereas the upregulated IL-β production in PAMs induced by PRRSV was mediated via TLR4/MyD88 pathway [49]. Possibly, the reduced IL-1β secretion in PGE by type 2 may be due to the suppressive effect of PRRSV on TLR4 expression.

It leads us to believe that the reduced TLR4/IL-1β system and TNF-α production by PRRSV might be the strategy of virus to escape the host cells destruction. Herein, the decreased CPE with persistence of PRRSV type 2 at 6 dpi may support these assumptions. Taken together, the downregulated TNF-α and IL-1β secretion by PRRSV in immune and PGE cells reflects a poor innate immune response which leads to secondary infection by other microbial pathogens [50].

Indeed, PRRSV type 2, but not type 1 infection, induced *IL-1β*, *IL-6*, *IL-8* and *TNF-α* mRNA expression at 4 dpi. This evidence was not in agreement with the decreased IL-1β and TNF-α secretion at 4 dpi. Since the cytokine secretion was determined as the accumulated concentrations of cytokines in media from 0 dpi to 4 dpi, the increased *IL-1β*, *IL-6*, *IL-8* and *TNF-*α mRNA expression at 4 dpi should be performed their secretion at time later than 4 dpi.

Secretion of CCL2 and IFN-γ by PGE was additionally demonstrated in this study; however, secretion of IL-10 and IFN-α was absence in all groups. Thus, the presence of these innate immunity-related molecules indicates the ability of PGE to interact with the viral pathogens and establish a major part of the innate immune response of endometrial cells.

Apart from immune function, pro-inflammatory cytokines play roles in pregnancy and parturition. The locally increased IL-1β and TNF-α from uterine infiltrated neutrophil are required for muscle contraction and cervical ripening during parturition [51]. However, application of IL-1β and/or TNF-α caused the dissolution of collagen fibers, stromal edema, and severe inflammation in the cervix of guinea pigs [52]. Exposure to or infusion of IL-1β and TNF-α caused the defects associated with peripartum intrauterine inflammation, abnormal lung development associated with bronchopulmonary dysplasia, and brain injury [51]. Thus, the modulation of cytokine synthesis and release by PRRSV infection in PGE affecting the reproductive infertility needs a further study.

## Conclusion

Non-infected PRRSV endometrial cells, which express many PRRSV mediators and TLRs, but not classical PRRSV receptors Sn and CD163, can be susceptible and response to PRRSV infection. PRRSV induces the upregulation of PRRSV mediators continuing viral detection in PRRSV-infected PGE. The increased IL-6 and the decreased TNF-α and IL-1β secretion were also results of PRRSV infection. However, types of TLRs and pro-inflammatory cytokines mRNA expression in response to PRRSV type 1 and type 2 were different. The understanding of the induction of a proper innate immune response in PGE may be efficient for resolving the productive failure produced by PRRSV persistence in herds.
